# Trends in Dual Antiplatelet Therapy Use for Neurointerventional Procedures for the Management of Intracranial Aneurysms

**DOI:** 10.3390/biomedicines11082234

**Published:** 2023-08-09

**Authors:** Benjamen M. Meyer, Jessica K. Campos, Jonathan C. Collard de Beaufort, Ivette Chen, Muhammad Waqas Khan, Gizal Amin, David A. Zarrin, Brian V. Lien, Alexander L. Coon

**Affiliations:** 1 College of Medicine, University of Arizona, Tucson, AZ 85724, USA; 2 Department of Neurological Surgery, University of California Irvine, Orange, CA 92868, USA; 3 Syracuse University, Syracuse, NY 13244, USA; 4 Harvard University, Cambridge, MA 02138, USA; 5 Carondelet Neurological Institute, St. Joseph’s Hospital, Tucson, AZ 85711, USA; 6 School of Medicine, University of California, Los Angeles, CA 90095, USA

**Keywords:** intracranial aneurysm, dual antiplatelet therapy, monotherapy, flow diverting stents, stent-assisted coiling, intracranial stenting

## Abstract

The use of periprocedural dual antiplatelet therapy (DAPT) has significantly evolved along with innovations in the endovascular management of intracranial aneurysms. Historically, aspirin and clopidogrel have been the most commonly employed regimen due to its safety and efficacy. However, recent studies highlight the importance of tailoring DAPT regimens to individual patient characteristics which may affect clopidogrel metabolism, such as genetic polymorphisms. In the present report, a systematic review of the literature was performed to determine optimal antiplatelet use with flow diverting stents, intracranial stents, intrasaccular devices, and stent-assisted coiling. Studies were analyzed for the number of aneurysms treated, DAPT regimen, and any thromboembolic complications. Based on inclusion criteria, 368 studies were selected, which revealed the increasing popularity of alternative DAPT regimens with the aforementioned devices. Thromboembolic or hemorrhagic complications associated with antiplatelet medications were similar across all medications. DAPT with ticagrelor, tirofiban, or prasugrel are effective and safe alternatives to clopidogrel and do not require enzymatic activation. Further clinical trials are needed to evaluate different antiplatelet regimens with various devices to establish highest-level evidence-based guidelines and recommendations.

## 1. Introduction

Interventional cardiology laid the foundation for current developments in endovascular neurosurgery. The early utilization of intra-arterial catheters for interventional purposes were seen in cardiac vasculature targets [1]. There has been a dramatic rise in neuroendovascular treatment modalities for various intracranial pathologies over the last few decades, including intrasaccular coil embolization, stent-assisted coil (SAC) embolization, and flow diverting stents (FDS) [2]. Coil embolization, which involves placing a coil into an aneurysm to promote thrombus formation, has significantly improved the outcomes of patients with cerebral aneurysms [3]. Parent lumen flow diversion devices, such as the Pipeline Embolization Device (PED), are used to treat complex brain aneurysms that are wide-necked, giant, or not amenable for coiling [4]. FDS work by diverting blood flow away from the aneurysm, which promotes occlusion of the aneurysm dome over successive months [3]. The implementation of these neurointerventional devices has dramatically changed the course of treatment for intracranial aneurysms.

Modern neurointerventional procedures require pharmacological intervention to minimize thromboembolic complications. Dual antiplatelet therapy (DAPT), which includes aspirin with another antiplatelet agent, was initially studied in multi-center randomized trials in cardiac patients [5,6,7,8]. While the number of studies specific to neurointervention are increasing, it remains common practice for neurointerventionalists to follow a similar DAPT regimen to cardiac literature [9]. The optimal dosing and duration of DAPT in neurointervention is still debated [9,10,11].

Common antiplatelet agents for DAPT include ticlopidine, clopidogrel, prasugrel, and ticagrelor. Ticlopidine was first used in the late 1990s, but was quickly replaced by clopidogrel due to the occurrence of thrombocytopenia and neutropenia [12]. Clopidogrel has variable responsiveness [13] due to polymorphisms of liver enzymes in the multi-step activation pathway of clopidogrel [14,15]. Due to variations in the liver enzymes CYP2C9 and CYP2C19, it has previously been described that up to 30% of patients fail to respond as intended to clopidogrel [16]. A promising alternative to clopidogrel, prasugrel, has less frequently mutated enzymes [17] and is better at reducing ischemic events [7,15]. Another antiplatelet medication, ticagrelor, is often utilized for patients with clopidogrel-resistance [18]. Although several studies have compared ticagrelor and prasugrel against clopidogrel, the safety results remain unclear. While some studies have found no difference between the three medications [19,20], others found prasugrel and ticagrelor to have higher postoperative bleeding rates [7,21]. Tirofiban is an intravenous glycoprotein IIb/IIIa inhibitor with a short half-life [22,23]. The literature is conflicted when comparing its efficacy to other glycoprotein IIb-IIIa inhibitors and with traditional DAPT [24,25]. A meta-analysis discussing the use of tirofiban versus oral DAPT following SAC in ruptured aneurysms found tirofiban to have lower rates of post-procedural bleeding and thromboembolic complications [26]. However, additional studies have found that tirofiban in combination with DAPT did not increase post-procedural complications [27,28]. The choice of DAPT regimen also varies with the presence of ruptured versus unruptured aneurysms. Use of single versus dual antiplatelet therapy for ruptured aneurysms with subarachnoid hemorrhage remains debated [10]. Further studies are required to delineate optimal antiplatelet use for neurointerventional procedures.

The aforementioned antiplatelet medications are crucial to reduce thromboembolic complications following cerebrovascular procedures. Platelets tend to aggregate around foreign metals, which are the main composition of stents and coils [29]. While there is an abundance of literature on minimizing thrombosis and intracranial hemorrhage following neurointerventional procedures, there are no articles that summarize the use of DAPT therapy with intracranial aneurysm treatment. Here, we collected and analyzed data on the safety of various DAPT combinations with FDS, intrasaccular devices, and SAC procedures.

## 2. Methods

**Figure 1:**
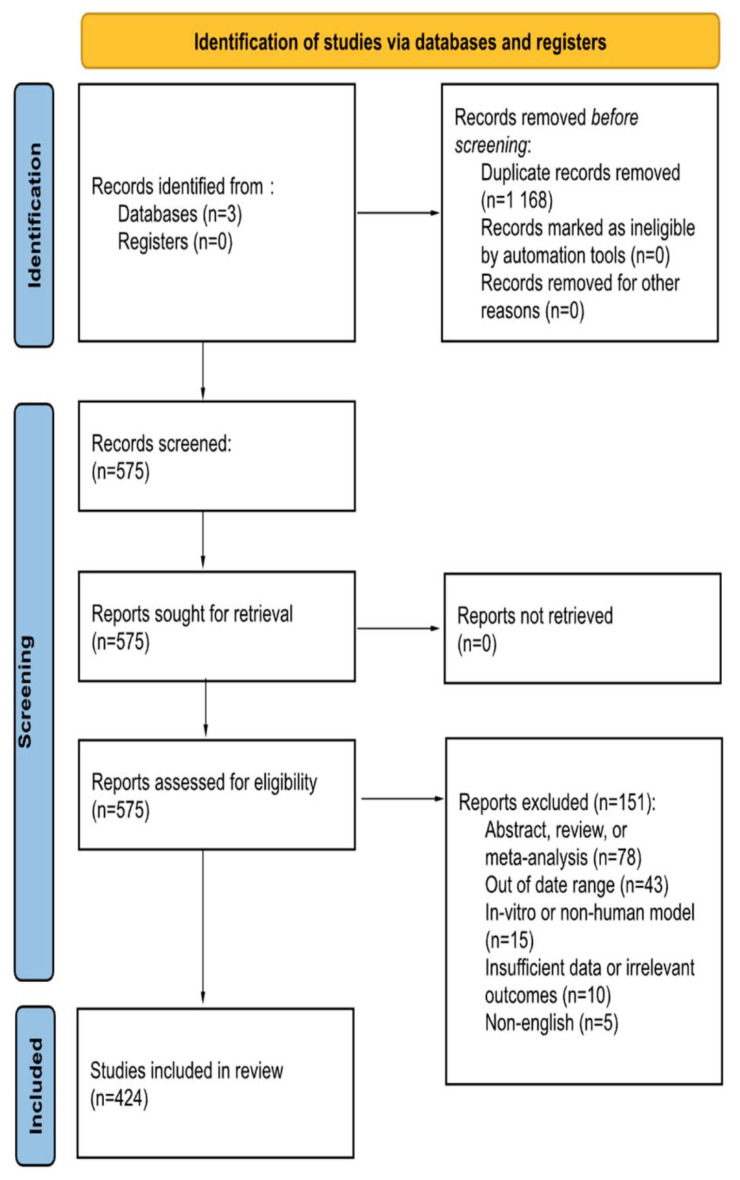
PRISMA Flow Diagram.

This study followed the Preferred Reporting Items for Systematic Reviews and Meta-Analyses (PRISMA) guidelines. The references for this systematic review were conducted using the Embase, Scopus, and PubMed databases on 10 May 2023 using a list of search phrases (Table 1). Information obtained includes: number of aneurysms treated, antiplatelet or anticoagulants used, length of medication use, in-stent stenosis, ischemic events, and hemorrhagic events (Figure 1). Studies were included if they reported on the use of DAPT with the treatment of intracranial aneurysms. Aneurysm management devices include flow diverting stents, stent-assisted coiling, and intrasaccular devices.

Exclusion criteria includes articles published in a non-English language, use of in-vitro or non-human models, and articles published outside of the date range. Manuscripts were excluded if they failed to report at least one of the following postprocedural complications: ischemic events, in-stent thrombosis or stenosis, and/or hemorrhage. Abstracts, including conference abstracts, literature reviews, systematic reviews, and meta-analyses were excluded. To conduct the review, each of the four independent reviewers screened each article using the title and abstract. Then, BMM, JCCdB, IC, and GA independently reviewed the full text for its content. Discrepancies were addressed by discussion with the authors. The articles identified were published between 1 January 2015 and 1 May 2023. Searches in PubMed returned 250+ articles, Embase returned 800+ articles, and Scopus returned 680+ articles. After applying the aforementioned exclusion criteria, a total of 368 unique articles were reviewed.

The objective was to determine the safety of DAPT use across various endovascular devices utilized for the endovascular treatment of intracranial aneurysms. Safety was defined by the rate of hemorrhagic, ischemic, and in-stent stenosis complications. Hemorrhagic complications included postoperative intracranial or extracranial hemorrhage. Ischemic, or thromboembolic complications, and in-stent stenosis were defined when present on imaging.

Percentages, odds ratios (OR), 95% confidence intervals (CI), and statistical significance were calculated using R software (R Foundation, Indianapolis, IN, USA). To compare the percentages and calculated *p*-Values, a *t*-test was used. The *p*-Value was considered statistically significant if less than 0.05. The rates of hemorrhagic, thromboembolic, and all complications were calculated per 100 cases within each device category and for each medication studied. For analysis, ischemic and in-stent stenosis were grouped into a single category. Studies that placed patients on more than one DAPT regimen were not included in the analysis to prevent confounding of the results. The use of various DAPT regimens were compared to one another for within each complication category using OR.

Review bias was minimized by having each of the four reviewers independently inspect each article using the inclusion and exclusion criteria as mentioned above. All excluded articles were reviewed by JKC to ensure there were none excluded due to bias. No automation tools or artificial intelligence software were used in this process.

## 3. Results

From 1 January 2015 to 1 May 2023, over 39,000 aneurysms across 368 studies and three types of procedures were included in this review.

### 3.1. Total Complications

**Figure 2:**
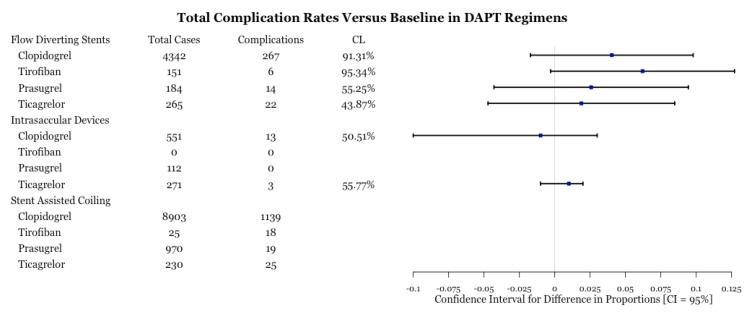
Test for difference of complication rates by drug type for three procedures. CL is the confidence level at which one can reject the null hypothesis of equality of a complication rate versus that of baseline. A higher CL is indicative that one of the drugs is more effective. Despite CL being high for tirofiban, there is a large margin of error due to a low number of complications.

**Figure 3:**
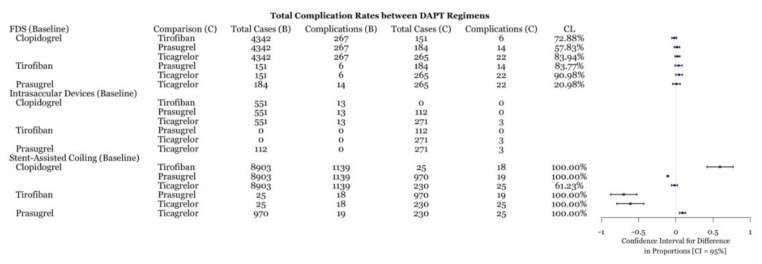
Difference of complication rates test between DAPT drugs for three procedures with associated confidence level.

A total of 1551 complications were reported for 16,783 aneurysms treated through three different methods: flow diversion, intrasaccular devices, and stent assisted coiling. For each of these methods, the number of baseline cases were 108 aneurysms (11 complications), 666 aneurysms (12 baseline), and five aneurysms (two complications). Figure 2 provides a breakdown of the total number of cases and complication events per drug type. In flow diverting procedures, we are 95.34% and 91.31% confident that tirofiban and clopidogrel, respectively, had significantly lower complication rates than baseline treatment. Tirofiban and clopidogrel have similar complication rates between each other (Figure 3). The use of clopidogrel and ticagrelor in intrasaccular device procedures showed comparable complication rates (Figure 3).

### 3.2. Hemorrhagic Complications

**Figure 4:**
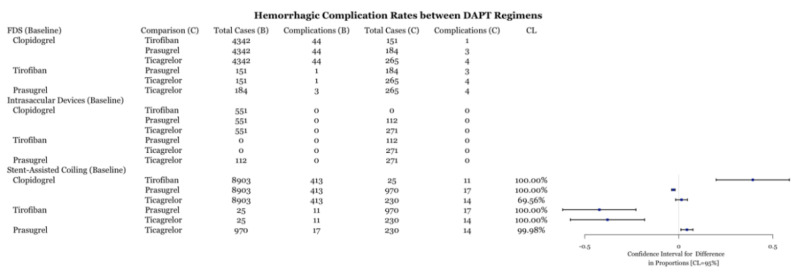
Difference of hemorrhagic complication rates between DAPT drugs for three procedures with associated confidence levels.

Reported postoperative hemorrhagic complications were compared between the four drug types for each procedure. FDS and intrasaccular devices did not have enough cases for any statistical analysis (n < 5). For stent-assisted coiling, hemorrhages were reported in 413 out of 8903 aneurysms using clopidogrel, 11 out of 25 for tirofiban, 17 out of 970 with prasugrel, and 14 out of 230 for ticagrelor. In terms of hemorrhagic complication rates, clopidogrel had statistically fewer complications than tirofiban, similar complication rates to ticagrelor, and more complications than prasugrel (Figure 4). Both prasugrel and ticagrelor had statistically lower complication rates than tirofiban (Figure 4). Prasugrel and ticagrelor exhibited similar total complication rates (Figure 2).

### 3.3. Ischemic and Thrombotic Complications

**Figure 5:**
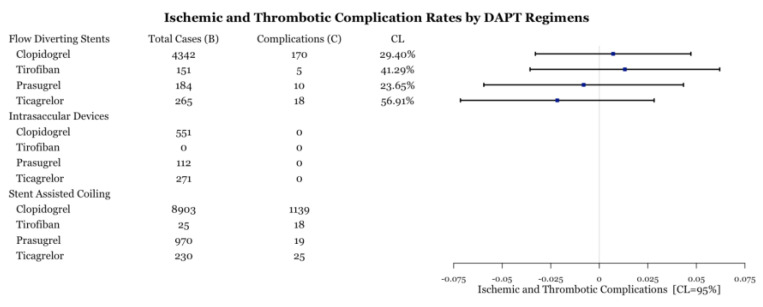
Test for difference of complication rates by drug type for three procedures. CL is the confidence level at which one can reject the null hypothesis of equality of a complication rate versus that of baseline.

**Figure 6:**
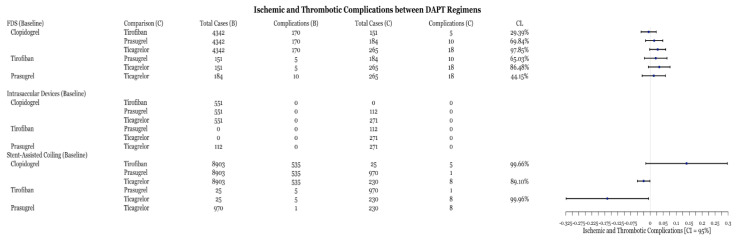
Difference of ischemic and thrombotic complication rates between DAPT drugs for three procedures with associated confidence levels.

Within each medication category, the number of ischemic and thrombotic complications were compared between medication types. All intrasaccular device procedures and stent-assisted coiling procedures with prasugrel did not have enough cases (n < 5) for statistical analysis (Figure 5). All medications performed similarly within flow diversion cases based on the value of the point estimate; however, the confidence level is low for most comparisons (Figure 5). Within stent-assisted coiling cases, ticagrelor had significantly fewer complications than both prasugrel and clopidogrel (Figure 6). Despite the point-estimate being high, there is statistically no difference in complication rates between clopidogrel and tirofiban since the confidence interval includes 0 (Figure 6). Ticagrelor had significantly lower complication rates than tirofiban (Figure 6).

## 4. Discussion

### 4.1. General DAPT Use for Aneurysm Treatment

DAPT use in endovascular aneurysm treatment has witnessed significant advancements and refinements since the popularization of intracranial stenting in the early 1990s [30]. Antiplatelet medications have been employed in combination to optimize the prevention of thrombotic events after neurointerventional procedures [31]. Aspirin, a widely used antiplatelet agent, acts by irreversibly inhibiting cyclooxygenase-1, thereby suppressing platelet aggregation [32]. Clopidogrel, another common antiplatelet medication, is a thienopyridine derivative that selectively inhibits adenosine diphosphate-induced platelet activation by binding to the P2Y12 receptor [33]. The combination of aspirin and clopidogrel has historically been the most commonly employed DAPT regimen in unruptured aneurysm treatment due to its safety and efficacy [34]. However, a fixed-dose combination was frequently used without consideration of individual patient variability [35]. Emerging evidence has highlighted the importance of tailoring DAPT regimens based on patient characteristics, such as genetic polymorphisms, that greatly affect clopidogrel metabolism and response [36]. A variety of assessments of antiplatelet responsiveness are available, including aggregometry and platelet reactivity assays, which have enabled clinicians to optimize the antiplatelet effect [37]. This personalized approach has led to the development of other antiplatelet agents, including tirofiban, prasugrel, and ticagrelor, which may exhibit more predictable and potent platelet inhibition [38]. Ongoing research efforts aim to elucidate the optimal duration of DAPT, evaluate the efficacy of new agents, and identify predictors of patient response to guide individualized management plans.

This meta-analysis demonstrates several important points. First, the comparison of individual DAPT regimens to each other for use with FDS demonstrated no difference in preventing ischemic or thrombotic complications (Figure 6). Thus, it may suggest that clopidogrel alternatives, including tirofiban, prasugrel, and ticagrelor, are as effective as clopidogrel at preventing thromboembolic complications. The SAC results demonstrate that ticagrelor was more effective at an 89% confidence level (Figure 6) at preventing ischemic and thrombotic complications than clopidogrel. Clopidogrel was more effective at reducing these complications at an 99.66% confidence level (Figure 6). However, the inclusion of zero in the confidence level indicates a low but statistically significant sample size, which necessitates further data collection. These results support the placement of patients that are hypo-responders to clopidogrel on ticagrelor or using intravenous tirofiban. Likewise, prasugrel was demonstrated to be more effective at preventing all complications in SAC compared to clopidogrel (Figure 3). Examination of the hemorrhagic complications demonstrates that prasugrel is more effective, and ticagrelor as effective, at preventing intracranial hemorrhagic complications with SAC than clopidogrel (Figure 4). Alternatively, clopidogrel was shown to be more effective at preventing intracranial hemorrhagic complications in comparison to tirofiban when used with SAC (Figure 4). While the risk and extent of intracranial bleeding in the management of aneurysms increases with DAPT, more data are required to compare the effectiveness of various DAPT regimens in preventing intracranial hemorrhagic complications in FDS and intrasaccular devices. Randomized clinical trials are required to compare the effectiveness of various DAPT regimens against each other.

### 4.2. DAPT Use in Stent-Assisted Coiling

Although the combination of aspirin and clopidogrel is historically popular in aneurysm treatment, other agents are increasing in use for SAC. A novel and increasingly favored medication, ticagrelor, has gained popularity in DAPT for SAC due to its more predictable and potent platelet inhibition as compared to clopidogrel [39]. Similar to tirofiban, it has a rapid onset of action and does not require metabolic activation [40]. This decreases the risk of unresponsiveness, which may be seen with clopidogrel use [41]. Across 130 unruptured aneurysms treated with SAC and managed with aspirin and ticagrelor, Ma et al. reported thromboembolic complications in 3.3% of patients, and 1.3% of patients developed extracranial hemorrhage [39]. Narata et al. reported three (1.9%) ischemic and six (3.9%) intracranial hemorrhagic complications following treatment of 154 unruptured aneurysms with FDS and SAC that were on DAPT with ticagrelor [42]. While some have proposed that ticagrelor has an increased risk of intracranial hemorrhagic complications, Yi et al. demonstrated similar thromboembolic complications between ticagrelor and clopidogrel in patients managed with SAC for unruptured aneurysms [43]. A potential disadvantage of ticagrelor is its short half-life, which necessitates twice-daily dosing [44]. This necessity may make the medication dangerous for patients with a history of noncompliance or nonadherence [44].

An increasingly popular oral antiplatelet agent is prasugrel, which has been demonstrated to have lower ischemic events than clopidogrel in acute coronary syndrome [45]. A recent comparative study of DAPT with clopidogrel versus prasugrel in SAC for unruptured aneurysms revealed less thromboembolic complications with similar intracranial hemorrhagic events in the prasugrel group [46]. Likewise, the current study demonstrated lower rates of all complications with prasugrel versus clopidogrel use (Figure 3).

Although rare, the use of aspirin as monotherapy has been shown to be effective in SAC. A multicenter study on antiplatelet use in SAC demonstrated nonsignificant differences in the rates of ischemia and intracranial hemorrhage between aspirin monotherapy and DAPT in ruptured aneurysms [47]. While this supports the use of single antiplatelet therapy (SAPT) in SAC, this study was small and there has been another report of thromboembolic complications with this regimen in ruptured aneurysms [48]. Further studies are required to determine the safety and efficacy of SAPT in SAC.

Lastly, tirofiban is an intravenous GIIb/IIIa antagonist with a rapid onset of action, which makes it particularly useful in SAC procedures that require immediate platelet inhibition [27]. This medication may also be used in the setting of acute stent implantation, ineffective DAPT, or in combination with oral agents to enhance treatment [27,49]. Its efficacy in preventing thromboembolic events is well documented in the literature and may even be useful as inpatient monotherapy [49]. For ruptured aneurysms managed with FDS or SAC, a DELPHI consensus study determined that DAPT with tirofiban or eptifibatide was used as a standard approach amongst the responders [50]. In a case series of 105 patients with ruptured aneurysms who underwent SAC with prophylactic tirofiban, 2.8% of patients had thromboembolic complications without new intracranial hemorrhage [27,51]. This is further supported by a study on SAC and FDS by Samaniego et al., which found that tirofiban with DAPT did not increase the rate of thromboembolic or intracranial hemorrhagic complications in both unruptured and ruptured aneurysms [27]. A recent study found there to be thromboembolic complications in 13.89% and 8.33% of patients on DAPT for treated ruptured aneurysms with clopidogrel versus tirofiban, respectively [52]. Furthermore, intracranial hemorrhage was only found in 1.39% of patients in the tirofiban group. A recent meta-analysis and case series found there to be significantly lower rates of thromboembolic complications without an increase in intracranial hemorrhage when prophylactic therapy with tirofiban versus DAPT was used in SAC for ruptured aneurysms [26,53]. A recent meta-analysis demonstrated lower intracranial hemorrhagic and thromboembolic complications in patients managed with SAPT with tirofiban or eptifibatide over clopidogrel/aspirin in ruptured and unruptured aneurysms [54]. The current study of 114 articles supports these results, demonstrating that ticagrelor and prasugrel were more effective and tirofiban as effective at preventing thromboembolic complications compared to clopidogrel (Figure 6). However, a disadvantage of tirofiban is its short half-life, thus careful monitoring to maintain optimal antiplatelet effect is necessary [55]. Additionally, it can only be given intravenously, making it restricted to inpatient use [56]. Overall, tirofiban is the preferred antiplatelet medication for ruptured aneurysms, while various combinations of DAPT may be used for unruptured aneurysms.

When considering the prevention of thromboembolic events in SAC for aneurysm treatment, an individualized patient approach should be taken. A recent study demonstrated the efficacy of thromboelastography-platelet mapping in stent-assisted coiling for ruptured aneurysms, which would allow for an individualized antiplatelet regimen [57]. This would help prevent thromboembolic complications from occurring in those with antiplatelet resistance [58]. The choice for DAPT in SAC should consider the patient’s risk of bleeding, history of noncompliance, and individual drug metabolism.

### 4.3. DAPT with FDS and Aneurysm Treatment

While the use of clopidogrel for DAPT in flow diversion is well established, there has been a movement towards the use of various other antiplatelet regimens. The current study recognized the use of antiplatelet therapies across 14,099 cases over 206 studies. There was no statistically significant difference between the various DAPT regimens and the rate of thromboembolic and/or intracranial hemorrhagic complications (Figure 3 and Figure 4). These results suggest that clopidogrel alternatives are safe antiplatelet medications for use in FDS. Several of the original PED, a type of flow diverter, trials used aspirin and clopidogrel to prevent thrombotic complications after device placement in ruptured aneurysms [59]. For example, the PUFS trial placed patients with unruptured aneurysms on aspirin 325 mg and clopidogrel 75 mg once daily [60]. However, as discussed previously, clopidogrel is a prodrug that must first be converted to its active metabolite by a CYP liver enzyme [14]. This creates the possibility of hyper- and hypo-responsiveness due to genetic variations and concurrent medication use that inhibits or activates CYP [61]. As such, there has been a shift towards tirofiban, ticagrelor, or prasugrel with aspirin. Previous studies comparing aspirin and clopidogrel versus aspirin and aspirin and ticagrelor found no significant difference in intracranial hemorrhagic or thromboembolic complications in the management of unruptured aneurysms [62]. A recent meta-analysis reported not only the safety of ticagrelor or prasugrel with DAPT, but it found ticagrelor to be associated with reduced mortality in comparison to clopidogrel in unruptured and ruptured aneurysms [63]. Furthermore, the use of prophylactic tirofiban with conventional DAPT for unruptured aneurysms, being clopidogrel with aspirin, was found to have lower rates of thromboembolic complications as compared to only DAPT [64]. Although these studies are not without their limitations, they suggest that more effective medications exist for commonly treated conditions.

Within the past few years, there have been reports of using triple therapy (TT), which is DAPT with oral anticoagulation. In a case series, Siddiqui et al. compared the use of DAPT and TT in patients with unruptured vertebrobasilar fusiform aneurysms [65]. With a similar number of FDS with adjunctive coiling placed between the two treatment groups, they reported the TT group to have fewer strokes and better overall outcome [65]. However, there was a recent case report of an unruptured aneurysmal recanalization after TT, with subsequent aneurysm re-occlusion after stopping the oral anticoagulant [66]. On a similar topic, Wu et al. reported the periprocedural use of tirofiban with conventional DAPT and found it to be significantly more effective at preventing thromboembolic events in unruptured aneurysms without an increase in intracranial hemorrhages [64]. Further studies are warranted to determine the use, efficacy, and safety of TT in flow diversion.

To minimize the requirements for DAPT, a new FDS with a hydrophilic polymer coating (p48-MW-HPC) has been developed. This has a similar goal to the novel PED-Shield: a FDS with a phosphorylcholine coating designed to reduce thrombogenicity [67]. Either of these devices may be preferred in cases of ruptured aneurysms due to the reduced need for antiplatelet agents [67]. Lobsien et al. demonstrated the effectiveness of the p48-MW-HPC FDS under SAPT across 13 ruptured aneurysms and reported a single thromboembolic complication [68]. A small cohort study demonstrated the limitations of the device with monotherapy with ruptured aneurysms, reporting intraprocedural thrombus formation in 50% of patients, postprocedural thrombus in 12.5% of patients, and vasospasm in 25% of patients [69]. Preliminary animal model studies found there to be significantly more thrombus formation in the PED-Shield group on aspirin versus the DAPT group [70]. A recent meta-analysis on ruptured and unruptured aneurysms demonstrated SAPT with aspirin resulted in significantly more thromboembolic and intracranial hemorrhagic complications than SAPT with prasugrel or ticagrelor [71]. However, there have been successful reports of aspirin monotherapy in flow diversion for unruptured aneurysms in patients with bleeding disorders [72]. Further studies and large clinical trials are required to determine the effectiveness of coated FDS when used with monotherapy or without any antiplatelet agent.

### 4.4. DAPT with Intrasaccular Devices and Aneurysms

The development of intrasaccular flow disruptors has limited the need for concurrent need for DAPT. These devices are metal mesh spheres that work by volumetrically filling the aneurysm, thus achieving roles as both a flow diverter and coil [73]. One such device, the Woven EndoBridge (WEB) (Microvention, Aliso Viejo, CA, USA), has been demonstrated to be effective in ruptured aneurysms and may only require SAPT with aspirin [74]. WEB is currently the only intrasaccular device approved in the USA and is indicated for use in wide-necked bifurcation aneurysms [73]. If the entire WEB device is within the aneurysm, antiplatelet therapy may not be required at all [73]. If the mesh components protrude into the parent vessel, this poses a risk for thrombosis and antiplatelet medications are recommended [73]. A recent systematic review about WEB use in ruptured and unruptured aneurysms by Xie et al. found 1/7 articles reported any antiplatelet use [75]. This review found thromboembolic complications to occur in 7% of the collected cases [75]. The current study found 48 studies with 3583 cases that used an intrasaccular device for the management of intracranial aneurysms. Although, there was not enough data to suggest an advantage of one DAPT regimen over another for preventing thromboembolic or intracranial hemorrhagic complications (Figure 5 and Figure 6). The lack of data may be attributed to the device’s novelty or the diminished requirement for DAPT therapy and thus additional comparative studies are warranted. The Nautilus Intrasaccular System (EndoStream Medical, Or Akiva, Israel) is a novel intrasaccular device unavailable in the USA that has demonstrated low complication rates without the need for antiplatelet therapy in ruptured aneurysms [76]. The novel ARTISSE (Medtronic, Irvine, CA, USA) intrasaccular device, which is also not available in the USA, demonstrated thromboembolic complications in two out of the nine unruptured cases performed [77]. These patients were placed on antiplatelet therapy to prevent further strokes. While current intrasaccular devices have demonstrated efficacy, further studies are required to determine their safety in the absence of any antiplatelet therapy.

### 4.5. Limitations

The nature of this article creates inherent limitations. For one, there is still bias present when choosing articles to include even with proper checks and balances. Furthermore, the use of three databases limits the scope of articles that may be reviewed. The inclusion of case reports and series may have skewed the data because these articles are written in the setting of unique pathology or technique which may not be applicable to the general population. The search phrases “intrasaccular device” and “stent-assisted coiling” are limited because not all published works include these phrases in their title or abstract, thus limiting the amount of articles included. Similarly, “dual antiplatelet therapy” may not have been mentioned in the title or abstract if it was not the focus of the study. The use of this review for DAPT use in practice should be approached with caution. Randomized controlled trials of all medications across each device use should be performed to provide a more clear view of recommended DAPT regimens. Ultimately, it remains the physician’s choice and experiences to determine the best DAPT regimen for the individual patients.

### 4.6. Future Directions

Comparison studies and clinical trials that compare the efficacy and safety of the various antiplatelet regimens employed for the different aneurysm treatment strategies are warranted. Furthermore, studies that examine the optimal duration of antiplatelet use, which balance the risk of thrombus versus intracranial hemorrhagic complications, are justified.

## 5. Conclusions

While the use of DAPT in the treatment of intracranial pathology is standard, the optimal regimen remains unclear. Aspirin with clopidogrel has become a mainstay for DAPT, although varied clopidogrel responses have led to the popularization of prasugrel, tirofiban, ticagrelor, and cilostazol. If platelet function resting reveals an effective response to clopidogrel, trends in the literature and practice patterns suggest an antiplatelet therapy with clopidogrel or prasugrel given their safety profiles and low cost. In the event of an ineffective response to clopidogrel, oral prasugrel or ticagrelor with or without intraoperative tirofiban has been a developing practice pattern in modern neurointervention. The out-of-pocket cost to patients should be considered when prescribing the aforementioned second generation antiplatelets, as medication compliance is paramount to preventing post-embolization thrombotic complications. The use of devices with antiplatelet coatings is a promising advancement towards monotherapy; however, initial results suggest that DAPT may still be required to reduce thromboembolic complications. Randomized clinical trials of SAPT, DAPT, and TT use during the management of intracranial pathologies is necessary to delineate the optimal treatment regimen.

## Figures and Tables

**Table 1:** Search phrases with their numerical results.

Search Phrase	PubMed	Embase	Scopus
(dual antiplatelet therapy) AND (flow diversion)	95	220	197
(dual antiplatelet therapy) AND (pipeline embolization device)	70	230	184
(dual antiplatelet therapy) AND (fred)	7	47	32
(dual antiplatelet therapy) AND (p64)	3	8	6
(dual antiplatelet therapy) AND (silk)	2	29	24
(dual antiplatelet therapy) AND (surpass)	6	31	32
(dual antiplatelet therapy) AND (stent-assisted coiling)	59	174	158
(dual antiplatelet therapy) AND (intrasaccular device)	3	15	9
(dual antiplatelet therapy) AND (WEB)	8	51	43
Total	253	805	685

## Data Availability

Not provided.
